# Assessment of *Angelica sinensis* (Oliv.) Diels as a repellent for personal protection against mosquitoes under laboratory and field conditions in northern Thailand

**DOI:** 10.1186/s13071-016-1650-y

**Published:** 2016-06-29

**Authors:** Danita Champakaew, Anuluck Junkum, Udom Chaithong, Atchariya Jitpakdi, Doungrat Riyong, Anchalee Wannasan, Jitrawadee Intirach, Roongtawan Muangmoon, Arpaporn Chansang, Benjawan Tuetun, Benjawan Pitasawat

**Affiliations:** Department of Parasitology, Faculty of Medicine, Chiang Mai University, Chiang Mai, 50200 Thailand; Department of Food Industry and Service, School of Culinary Arts, Suan Dusit Rajabhat University Lampang, Lampang, 52000 Thailand

**Keywords:** *Aedes aegypti*, *Angelica sinensis*, Dong quai, DEET, Mosquito repellency, Field repellent

## Abstract

**Background:**

*Angelica sinensis* (Oliv.) hexane extract (AHE) has been reported as a proven and impressive repellent against laboratory-reared female *Aedes aegypti* mosquitoes. With the aim of promoting products of plant origin as a viable alternative to conventional synthetic substances, this study was designed to transform AHE-based repellents for exploitable commercial production by enhancing their efficacy and assessing their physical and biological stability as well as repellency against mosquitoes under laboratory and field conditions.

**Methods:**

The chemical profile of AHE was analyzed by qualitative gas chromatography-mass spectrometry (GC-MS) technique. AHE was supplemented with vanillin, as a fixative, and then investigated for repellency and comparison to the standard synthetic repellent, DEET, under both laboratory and field conditions. Determination of physical and biological stability as a repellent was carried out after keeping AHE samples under varying temperatures and for different storage times.

**Results:**

GC-MS analysis revealed that AHE contained at least 21 phytochemical compounds, constituting 95.74 % of the total content, with the major constituent of 3-*N*-butylphthalide (66.67 %). Ethanolic formulations of AHE and DEET showed improvement of repellency in a dose-dependent manner when vanillin was added in laboratory assessment. While 5–25 % AHE alone provided median complete-protection times of 2.0–6.5 h against *Ae. aegypti*, these times were increased to 4.0–8.5 h with a combination of AHE and 5 % vanillin (AHEv). Protection times against *Ae. aegypti* were extended from 2.25 to 7.25 h to 4.25–8.25 h when 5–25 % DEET was combined with 5 % vanillin (DEETv). In determining stability, all stored AHE samples exhibited similar characteristics such as liquid phases with aromatic odor comparable to those of fresh preparations. Furthermore, repellent activity of stored AHE samples lasted for at least six months, with varied efficacy (4.5–10.0 h) against *Ae. aegypti*. Field trials revealed strong repellency from both 25 % AHEv and 25 % DEETv, with complete protection (100 %) against a wide range of local mosquito populations. A total of 5,718 adult female mosquitoes, with the most predominant being *Culex quinquefasciatus* (41.47 %), *Armigeres subalbatus* (41.13 %), and *Culex vishnui* (10.53 %), was collected during field applications. No local skin reaction or other allergic responses was observed during both laboratory and field study periods.

**Conclusions:**

*Angelica sinensis* proved to have not only impressive repellency against both laboratory *Ae. aegypti* and a wide range of natural mosquito populations, but also relative stability in physical and biological performance.

## Background

Mosquito control and personal protection from mosquito bites are the most meaningful measures for controlling several life-threatening diseases such as dengue fever, yellow fever, malaria, filariasis and Japanese encephalitis, which are transmitted solely by bites from bloodsucking mosquitoes [1, 2]. While mosquito control programs can reduce the rates of vector-transmitted diseases, the first line of defense continues to be various personal protective methods, for example, avoiding known mosquito habitats and peak biting times, wearing protective clothing, and using bed nets and insect repellents [3–5]. Repellent application has long been seen to minimize human contact with vector and nuisance insects, and is one of the most reliable means of personal protection against annoyance and pathogenic infections. Use of repellents seems to be a simple, practical and economical approach to prevent mosquito-borne diseases not only for local people, but also for travelers in disease risk areas, particularly in tropical countries [6–9]. The chemical compound, *N,N*-diethyl-3-methylbenzamide (formerly known as diethyl-*m*-toluamide and commonly called DEET), is considered the best mosquito repellent developed so far and has been used as a “gold standard” to which new candidate repellents are compared [10, 11]. Currently, DEET is the most ubiquitous active ingredient used in commercially available repellents, with remarkable effectiveness in protecting against mosquitoes and other biting insects [1, 12]. Despite the widespread use of commercial formulations containing DEET and other synthetic substances, the search for alternative repellents has become more important, due to increasing concerns about synthetics-related problems such as unpleasant odor and possible health risks associated with repeated use or misapplication [1, 13].

During recent years, global attention has been paid towards exploring natural repellents, particularly those of herbal origin. A variety of plant products, derived from members of the families, Asteraceae, Cupressaceae, Labiatae, Lamiaceae, Lauraceae, Meliaceae, Myrtaceae, Piperaceae, Poaceae, Rutaceae, Umbelliferae and Zingiberaceae, have been evaluated for repellency against various mosquito vectors, but few compounds have been exploited commercially [3, 14–17]. Although products that derived from plants are promoted occasionally as safer and suitable alternative repellents, most of them are produced and distributed locally, and appear on the market for only a short time [18, 19]. Furthermore, many studies have shown that almost all registered commercial products containing botanical active ingredients only offer limited protection and require more frequent reapplication than even a low concentration of DEET-based repellents [5, 18–20]. However, the growing demand for natural alternative repellents in the community illustrates further the need to evaluate new plant-based products critically for personal protection against mosquitoes and mosquito-borne illnesses [3, 19].

**Fig. 1:**
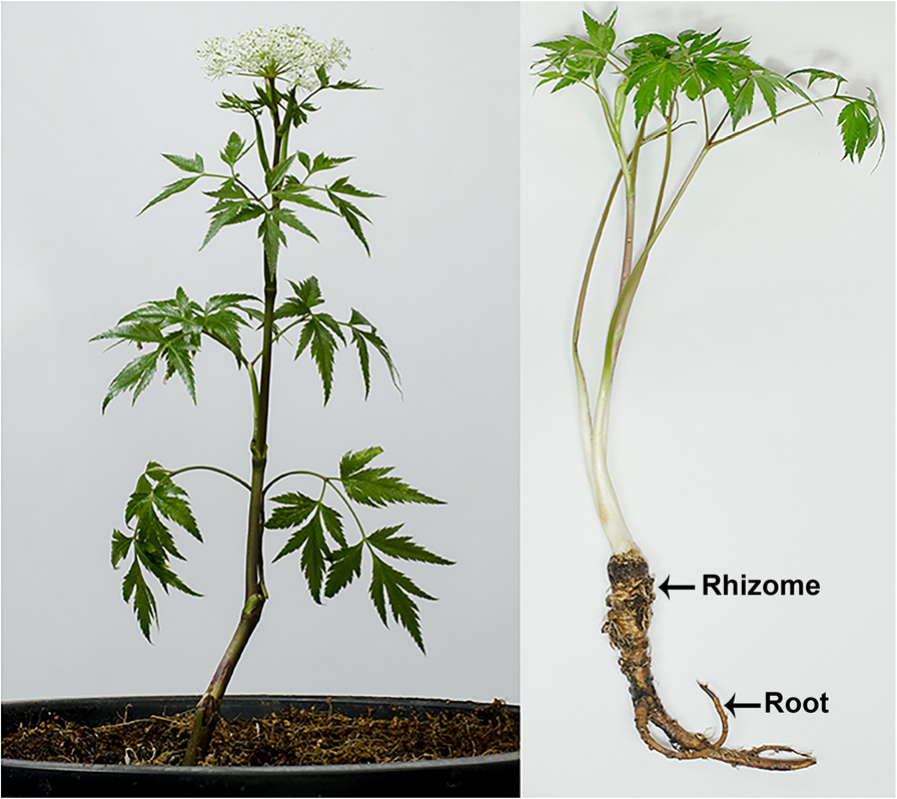
*Angelica sinensis* (Oliv.) Diels (Umbelliferae), with rhizome and root marked by arrows

In the ongoing research that promotes products of plant origin for protection against mosquitoes and other haematophagous insects, *Angelica sinensis* (Oliv.) Diels (Fig. 1) is a promising plant that has attracted substantial interest. Preliminary repellent assessments of various plant products, essential oils and solvent extracts, against laboratory-reared female *Aedes aegypti* mosquitoes highlighted *A. sinensis* hexane extract (AHE) as offering the longest-lasting protection time of 7.5 (6.5–8.5) h [21]. Phthalides, including 3-*N*-butylphthalide (70.14 %), ligustilide (8.34 %) and butylidenephthalide (4.85 %) were reported as the main and minor constituents in AHE, respectively. No local skin reaction such as rash, irritation, hot sensation or swelling was observed in AHE-applied volunteers during the study period [21]. Interestingly, *A. sinensis* known commonly as dong quai (English) and dang gui (Chinese), the name of its root, has a history of medical application. It has been used for thousands of years in traditional Chinese, Japanese and Korean medicine as a general blood tonic and treatment for heart, lung, liver, spleen, intestinal and gynecological disorders [11, 22, 23]. Dong quai is used generally as a flavoring agent in food, tea and tincture period. Its tea and tincture are effective in helping to normalize female hormones, ease arthritic pain and lower blood pressure [24, 25]. Regarding its anti-insect properties, the essential oil of *A. sinensis* and its constituents were found to possess repellent activity against mosquitoes and the German cockroach, *Blatella germanica* [11, 26]. Also, (Z)-ligustilide, a principal compound derived from *A. sinensis* root oil was reported as a potent deterrent for mosquito biting and feeding in both *Ae. aegypti* and *Anopheles stephensi* mosquitoes [11]. Consequently, *A. sinensis* is considered worthy of further development as an alternative repellent to conventional synthetic substances, particularly DEET, for protection against mosquitoes.

In order to transform AHE-based repellents for exploitable commercial production, efficacy enhancing by synergistic combination and stability assessment in the physical and biological performance of AHE are topics of interest. Accordingly, one of the important developmental steps leading to the practical use of *A. sinensis* involves its application in the field, which yields information on repellent response in a wide range of mosquito species. Therefore, this study was designed to investigate the repellency of various AHE and DEET formulations incorporated with vanillin, a well-known fixative, against *Ae. aegypti* under laboratory conditions. The most effective AHE preparation was investigated for repellent activity and compared to DEET against natural mosquitoes under field conditions. Physical and biological performance of AHE as a repellent also was determined after keeping samples under varying temperatures and for different storage times.

## Methods

### Plant material and extraction procedure

Rhizomes and roots of *A. sinensis* were obtained commercially from Phakhinai Industry, Chiang Mai Province, northern Thailand. These plant materials were then identified morphologically and authenticated at the Department of Pharmaceutical Science, Faculty of Pharmacy, Chiang Mai University (CMU), Chiang Mai Province. A reference voucher, PARA-AN-003-Rh-Ro/1, was deposited at the Department of Parasitology, Faculty of Medicine, CMU. Shade-dried *A. sinensis* powder was extracted successively by macerating with hexane solvent for approximately 7 days at room temperature (27 ± 5 °C), with intermittent stirring. After vacuum filtration, the combined filtrates were concentrated until dry by using a vacuum rotary evaporator (EYELA, Tokyo, Japan) at 45 °C and then freeze-dried at -55 °C (Lyotrap Freeze Dryers) to obtain a liquid residue, which was brown with a strong aromatic odor. The resulting *A. sinensis* hexane extract (AHE) was stored in air-tight brown bottles in a freezer at -20 °C until its use in subsequent experiments.

### Gas chromatography-mass spectrometry (GC-MS) analysis

The chemical profile of AHE was determined by gas chromatography-mass spectrometry (GC-MS) analysis at the Science and Technology Service Center, CMU. This GC-MS system consisted of Hewlett-Packard GC 7890A Agilent Technology interfaced to a single quadrupole mass selective detector; MSD 5975C (EI) Agilent Technology. The column was a DB-5MS (30 m × 0.25 mm ID × 0.25 μm film thickness). The total GC-MS running time was 60 min. Helium was the carrier gas, set at a constant flow rate of 1.0 ml/min. The column temperature program was increased by 5 °C/min between 50 and 300 °C. The diluted sample (1/10 % v/v, in CH_2_Cl_2_) of 0.2 μl was injected manually in a split mode, with a 20:1 split ratio. The injector and detector temperatures were preformed at 250 and 280 °C, respectively. The mass spectra were operated in the electron ionization (EI) mode at 70 eV. Data were acquired over a range of 50–550 amu. The Kovats retention indices were calculated for all constituents using a homologous series of n-alkanes C_8_-C_40_ on DB-5MS column. Identification of individual components was done using the GC-MS (NIST 2008 and Wiley 8NO8) spectral libraries. The relative concentration of each compound in AHE was quantified according to the peak area integrated by the analysis program.

### Chemicals

The commercial chemical, *N,N*-diethyl-3-methylbenzamide (DEET: EC No. 2051497, Sigma-Aldrich, Saint Louis MO, United States), was used as the standard repellent. Vanillin (EC No. 2044652, Sigma-Aldrich, Buchs, Switzerland) is a fixative used in repellent formulations. All other chemicals and reagents used were of the analytical grade purchased from local agencies in Chiang Mai Province, Thailand.

### Mosquito rearing

Free-mating laboratory-reared *Ae. aegypti* mosquitoes, from a colony maintained since 1995 at the Department of Parasitology, Faculty of Medicine, CMU, were used in the repellent assessment of AHE ethanolic formulations as well as biological stability tests to determine the persistence of repellent activity in stored AHE samples. Mosquitoes were mass-reared using the modified measure of Limsuwan et al. [27] under controlled conditions of 27 ± 2 °C, 80 ± 10 % relative humidity (RH), and a 14:10 h light/dark photoperiod cycle, without exposure to pathogens or insecticides. Adults maintained in breeding cages were fed constantly with 10 % aqueous sucrose and 10 % v/v multivitamin syrup solution moistened on cotton pads. Blood meals from restrained albino rats were provided periodically to adult females for egg maturation. Eggs laid on filter paper soaked in plastic bowls of water were collected, allowed a few days to air-dry, and submerged in plastic trays filled with tap water for hatching. Freshly hatched larvae were fed daily with finely ground dog-biscuits. After pupation, pupae were transferred to plastic cups containing distilled water and subsequently placed in standard net cages (30 × 30 × 30 cm), where adults emerged. In order to induce blood feeding during repellent assessments, female mosquitoes (5 to 7 days after emergence) were starved by only being able to access cotton pads soaked with water for 12 h before testing.

### Human subjects

Healthy CMU graduate students of either sex, with no dermatologic or allergic reactions to mosquito bites or repellent applications, took an interview and were recruited as subjects for repellent assessment, as approved by the Research Ethics Committee of the Faculty of Medicine, CMU. Before conducting any human-related research procedures, all volunteers were briefed on the objective, methodology and nature of study participation, possible discomforts from exposure to test substances and mosquito bites, and remedial arrangements; and then they provided their written informed consent, which was deposited in the institute for future reference.

### Assessment on the repellent activity of AHE ethanolic formulations

AHE and DEET were formulated at various concentrations of 5–25 % in absolute ethanol with and without 5 % vanillin. Repellency investigation of the ethanolic formulations prepared from AHE and DEET against laboratory-reared female *Ae. aegypti* followed a modified version of the human-baited technique (arm-in-cage test) of the World Health Organization (WHO) standard guidelines [28]. Repellency testing was performed on *Ae. aegypti* in a 10 × 10 × 3 m room at 25–30 °C, 60–80 % RH, with exposure times at 08:00 and 18:00 h (normal feeding times for a day-biting mosquito). Prior to starting the experiment, 250 starved female mosquitoes aged 5–7 day-old were selected randomly, placed inside an experimental cage (30 × 30 × 30 cm), and left to acclimatize for 1 h. The arms of each volunteer were cleaned thoroughly with distilled water and air-dried. After that, each forearm was covered entirely with a plastic sleeve that had a 3 × 10 cm window exposing the skin on the ventral side, which acted as a test area. Disposable gloves were used to protect volunteers’ hands. The test formulation of 0.1 ml was applied thoroughly on the exposed skin of one arm, which performed as a treatment area, while the exposed skin on the other arm served as a control, with an equal volume of ethanol applied (with and without 5 % vanillin). To start the experiment, the control arm was placed inside the experimental cage and once two mosquitoes had bitten the exposed skin, the volunteer withdrew the control arm before blood feeding occurred. The repellency test was then continued by exposing the treated forearm in a similar manner. If no mosquitoes bit during a 3 min study period, the treated arm was removed from the cage and this test was repeated at 30 min intervals. During the experiment, successive introductions of the control arm before the treated arm were performed in order to standardize mosquito readiness to bite and nullify any bias. The experiment was complete once two mosquitoes had bitten on the treated site successively during a single exposure, or one had bitten once in each of two consecutive intervals. The complete protection time was defined as the duration between repellent application and the first two bites or two bites in successive intervals. Each experiment was duplicated using different batches of mosquitoes on different days for each of two human volunteers (one adult female and one adult male). No one tested more than one sample per day. Test samples and their order of testing were randomized in each volunteer, who was blinded to the repellent applied. Unpleasant odor of the test samples as well as skin irritation or other undesirable effects from each experiment also were observed and recorded.

### Determination of physical and biological stability of AHE

AHE samples were kept in conditions that varied in temperature (4 °C, ambient temperature and 45 °C) and storage time (one, three and six months). These samples were then subjected to comparison with fresh preparation for physical and biological performance. Physical characteristics and changes in appearance such as phase, color and odor of the stored AHE samples were observed at room temperature and recorded. Biological stability was determined from persistence of repellent activity, which was evaluated against laboratory female *Ae. aegypti* by using the modified human-treated measure of the WHO standard method, as described previously [28]. The repellent experiment was replicated twice on each of two volunteers (one adult female and one adult male).

### Field study area

A field trial for repellency against a natural population of mosquitoes was conducted from March to May 2013 at a residential site in a suburban area of Sunpesua subdistrict, Muang District, Chiang Mai Province (18°83′26″N, 09°00′15″E). This area of approximately 300 human habitations with trees, shrubs, grass, ponds and abundant mosquito breeding places such as ditches and sewage effluents, produced large populations of mosquitoes. While almost all inhabitants lived in houses with mosquito-screened doors and windows, domestic animals in this area such as dogs, cats and poultry, had no protection from insect bites and they probably served as a blood source for mosquitoes. Despite the rarity of vector-transmitted diseases in the area, documents based on a mosquito collection conducted in August 2003 [29] confirms that this location is suitable as a test site, due to its large and varied populations of mosquitoes, such as *Aedes gardnerii* (35.1 %), *Culex tritaeniorhynchus* (29.2 %), *Culex vishnui* (19.4 %), *Aedes lineatopennis* (5.1 %), *Armigeres subalbatus* (3.8 %) and *Mansonia uniformis* (2.3 %).

### Preliminary surveys

After obtaining a permission from the owner of this private land, preliminary human-baited-trap surveys were undertaken three times during the hot season from March to April in order to determine a suitable time for collecting mosquitoes. Pilot collections were performed for approximately 180–200 min, split into nine or ten 20-min periods between 17:00 and 21:00 h, in order that each of the three volunteers exposed to mosquitoes in natural field conditions received nine or ten biting collections. Mosquitoes landing on the exposed lower legs of CMU volunteers were captured before they imbibed blood by trained collectors, with the aid of a mouth aspirator and small flashlight. Mosquitoes collected from each volunteer in each period were placed into a screen-topped cup individually marked with the date, time of collection and collection site number. All collected mosquitoes were subsequently counted and identified to species under a stereomicroscope using the taxonomic keys of Tanaka et al. [30] and Rattanarithikul & Panthusiri [31].

### Field repellent bioassay

Field experimental trials were divided into two groups of volunteers, with each comprising two treated volunteers and a control individual. Each volunteer was protected by a jacket with hood, shoes with socks, gloves and long trousers rolled up to the knees, thus exposing only the lower part of the legs. Subjects deployed for collecting the mosquitoes that land on each exposed area were covered completely with outdoor clothes, gloves, and head mask. Of the three participants in each group, the two treated volunteers were given 2-ml aliquots of test samples topically and spread as evenly as possible on both lower legs from the base of the knee to the ankle. One treated volunteer was applied with 25 % AHE supplemented with 5 % vanillin (25 % AHEv) and the other was treated with 25 % DEET mixed with 5 % vanillin (25 % DEETv). An equal volume of 5 % vanillin in ethanol was applied in a similar manner on the lower legs of each control. After the treatment was applied, all of the volunteers were instructed not to wash until after the study time. Application of cosmetics, fragrances and body care products was avoided on the day of the assay. The two groups of volunteers were situated at least 20 m away from each other. The two repellent testers and one ethanol-treated control in each group sat on chairs about 5 m away from each other, while a mosquito collector sat opposite each volunteer. Exposure time was 180 min divided into nine 20-min periods between 18:00 and 21:30 h, in order that nine mosquito collections could be made on each subject. Mosquitoes landing on the exposed lower legs of treated and control volunteers were mouth aspirated by the collectors before the insects could imbibe any blood. After each 20-min period, the volunteers were moved to a new site at least 20 m from the last one, where they waited for 2 min before starting the next capture. The mosquitoes captured from each individual at each site were kept separately in labelled cups for counting and identifying under a stereomicroscope, using the taxonomic keys of Tanaka et al. [30] and Rattanarithikul & Panthusiri [31]. Data from the field assessments were analyzed to determine the number and species of mosquitoes collected during the exposure period, as well as the collection rate and percentage repellency provided by the test samples, as compared with the control. During the course of the study, volunteers, collectors and their positions were rotated randomly in order to prevent bias from any variations such as position and personal differences, which included the number of mosquitoes, catching ability, skin absorption and persistence of repellent, as well as attractiveness to the mosquitoes.

### Data management and statistical analysis

The median complete-protection time in the laboratory bioassays was used as a standard repellency criterion of the test samples against *Ae. aegypti*. Differences in significance were analyzed by a Kruskal-Wallis one-way ANOVA using SPSS 17.0 software. Statistically significant results were considered at *P* < 0.05. The effect of vanillin in prolonging the protection time of the test repellents was analyzed using the Mann-Whitney U Test. In analyzing field data, the total number of mosquitoes collected during each exposure was log-transformed before the mean and standard errors (SE) were analyzed. The Kruskal-Wallis one-way ANOVA also was used to determine the significance of difference between the controls and those volunteers treated at the critical level of 0.05. Percentage repellency (% Repellency) in the field trials was calculated by the following formula [32, 33]:

$$ \%\ \mathrm{Repellency} = \left(\mathrm{C}\hbox{-} \mathrm{T}\right)/\mathrm{C} \times 100 $$

where C is the number of mosquitoes collected from the lower legs of the controls and T is the number collected from the treated legs.

## Results and discussion

### Chemical composition of AHE

**Fig. 2:**
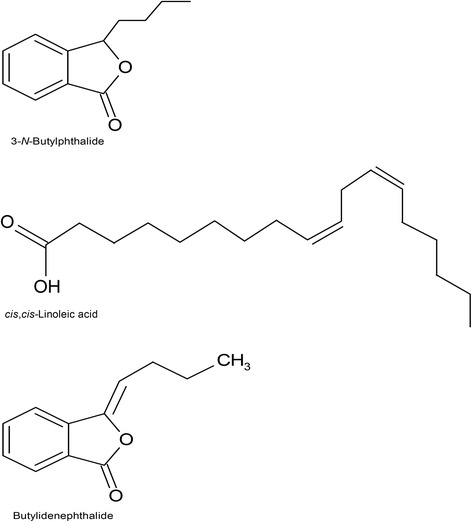
Chemical structure of the major and minor constituents of AHE

GC-MS characterization demonstrated that 21 phytochemical compounds were identified from AHE, accounting for 95.74 % of the total content (Table 1). The principal constituent was 3-*N*-butylphthalide (66.67 %), together with minor amounts of *cis*,*cis*-Linoleic acid (7.04 %) and butylidenephthalide (5.17 %). Chemical structures of these compounds are illustrated in Fig. 2. The percentage composition of the remaining 18 compounds ranged from 0.16 to 3.70 %. This GC-MS profile corresponded relatively to that of AHE analyzed in a previous study [21], which revealed the presence of 13 chemicals, representing 95.17 % of the total quantities. The most abundant compound was 3-*N*-butylphthalide (70.14 %), whereas ligustilide (8.34 %) and butylidenephthalide (4.85 %) were minor constituents. The remaining ten compounds ranged between 0.39 and 3.53 % of the total quantities. The chemical compositions, with phthalides predominating, of both AHE samples are quite compatible; this may be due to the similar extraction techniques from the same plant species, harvested from the same place in Chiang Mai Province, see [21].

**Table 1:** Chemical constituents of AHE

No	RT (min)	Compounds	Area (%)	KI^a^
1	8.86	*cis*-Ocimene	0.58	1038
2	15.43	6-Undecanone	0.19	1272
3	16.51	2-Methoxy-4-vinylphenol	0.34	1310
4	17.42	1,4-Cyclohexadiene-1,2-dicarboxylic anhydride	0.22	1346
5	19.41	*β*-Funebrene	0.52	1421
6	21.02	*β*-Chamigrene	0.16	1485
7	21.61	*β*-Bisabolene	0.38	1508
8	21.68	(+)-Cuparene	0.17	1511
9	23.31	Dehydroaromadendrene	0.81	1580
10	25.49	Butylidenephthalide	5.17	1675
11	27.24	3-*N*-Butylphthalide	66.67	1755
12	30.75	Palmitic acid methyl ester	0.80	1924
13	31.79	Palmitic acid	3.70	1978
14	33.95	Linoleic acid, methyl ester	1.68	2096
15	34.06	Oleic acid, methyl ester	0.20	2102
16	35.25	*cis*,*cis*-Linoleic acid	7.04	2163
17	41.30	Mono(2-ethylhexyl) phthalate	2.81	2528
18	46.17	1,9-Dioxa-4,6-diazacycloundecane-5-thione	0.58	2862
19	50.74	19-Methylene-5,10-secocholestan-3,5-dione	0.23	3208
20	51.11	Stigmasterol	0.82	3238
21	52.04	*β*-Sitosterol	2.67	3297
Total identified			95.74	

^a^Kovats index relative to *n*-alkane (C_8_–C_40_) on a DB-5MS column

### Repellent activity of ethanolic formulations

Repellent assessment of ethanolic formulations of AHE and DEET with and without 5 % vanillin supplementation demonstrated improved repellency in a dose dependent manner. While 5–25 % AHE alone provided median complete-protection times of 2.0–6.5 h against *Ae. aegypti*, the addition of 5 % vanillin increased AHE repellency (Z = -2.205, *P* = 0.029), with prolonged median complete-protection times of 4.0–8.5 h (Table 2). Vanillin also expanded the protection times of 5–25 % DEET against *Ae. aegypti* from 2.25 to 7.25 h to 4.25–8.25 h. Vanillin was selected in this study as an added fixative to improve the repellency of AHE, due to its noted ability in optimizing lasting quality in not only plant-based products, but also synthetic substances such as DEET [20, 34, 35]. Among a variety of commercial products are two categories of fixatives based on their sources: natural and synthetic materials [36, 37]. Natural fixatives can be derived from herbal constituents (vanillin, benzoin, myrrh, tolu balsam, etc.) and animal secretions (civet, castoreum, musk, ambergris, etc.). Vanillin (4-hydroxy-3-methoxybenzaldehyde) also can be derived from bioconversion of related natural products or synthesis. The synthetic fixatives used in repellent formations include glucam P-20, fixolide and 2,2,4-trimethyl-1,3-pentane diol. Fixative substances such as vanillin [20, 34, 35, 37, 38], mustard and coconut oils [39], liquid paraffin [40], salicyluric acid [41], and glucam P-20 and fixolide [37], improve repellent efficacy and are considered the simplest method when compared to other formulation techniques, such as microcapsule or nanoemulsion applications. Among fixative materials, vanillin has been preferable and selected widely as a synergistic additive in various mosquito repellents, with encouragingly improved efficacy. Songkro et al. [37] reported vanillin, a naturally fragrant fixative, as being the most effective in reducing the evaporation rate of citronella oil at 120 °C, when compared to synthetic compounds, such as glucam P-20 and fixolide. Consequently, vanillin was seen as the best fixative of citronella oil for effectively increasing the protective effect against *Ae. aegypti*. However, they suggested that besides the type and concentration of fixatives, the formula composition, such as ingredients incorporated into the preparations, also had some influence and played an important role in controlling repellent property. Correspondingly, Amer & Mehlhorn [42] stated that vanillin was not good enough to induce the same effect as a complex formulation (e.g. M10 containing 5 % of the five best oils at 1 % of each in ethanol and vanillin). Therefore, in the next step of developing AHE for exploitable commercial production, other fragrant fixatives, herbal active ingredients and additive materials would be incorporated. Methods of formulation used for amplifying repellency, particularly sustained-release technology that offers extended mosquito protection such as liposomes, microcapsules, nanoemulsions and nanosuspensions, also should be included. However, the formulation technique is important for not only increasing the effectiveness of a product, but also considering other factors such as health and economical aspects [42, 43]. These techniques appear to be valuable in producing AHE-based repellents with the concept of customer acceptability of a safe, cheap, convenient, practical and effective repellent.

**Table 2:** Repellent activity (median complete protection time: Median CPT) of the ethanolic formulations of AHE, AHEv (AHE + 5 % vanillin), DEET and DEETv (DEET+ 5 % vanillin) against female *Ae. aegypti*

% AHE or % DEET in the ethanolic formulations	Median CPT (Range, h)*			
	AHE	AHEv	DEET	DEETv
5 %	2.0 (2.0–3.5) a A	4.0 (3.0–4.5) a B	2.25 (1.5–2.5) a A	4.25 (3.5–6.0) a B
10 %	3.0 (2.5–3.0) a A	4.75 (4.5–5.0) ab B	3.0 (3.0–4.0) b A	5.0 (4.0–6.5) a B
15 %	4.0 (2.5–4.0) ab A	5.5 (4.5–6.5) bc B	6.0 (5.0–6.5) c B	7.5 (6.5–8.5) b C
20 %	4.75 (4.0–6.0) bc A	7.5 (7.0–7.5) d B	7.0 (6.0–7.0) cd B	8.0 (7.0–8.5) b B
25 %	6.50 (6.0–8.0) d A	8.5 (7.0–10.5) d A	7.25 (7.0–8.0) d A	8.25 (8.0–8.5) b A

*Values followed by different lowercase letters in a column and uppercase letters in a row are significantly different (*P* < 0.05)

### Physical and biological stability of AHE

The physical and biological performance of AHE samples, as determined after storage under 4 °C, ambient temperature (21 to 35 °C), and 45 °C for one, three and six months, showed little difference (Table 3). All stored AHE samples exhibited similar characteristics, liquid phases with aromatic odor, to those of the fresh preparation. However, the color changed from dark brown to very dark brown in samples kept at either ambient temperature for six months or at 45 °C for three and six months. These findings indicate relatively changeable appearance depending on the storing conditions of this product. However, the results obtained from testing these stored AHE samples against *Ae. aegypti* demonstrated that their repellent activity was present for a period of at least six months, with varied efficacy. Apart from the AHE samples kept at 4 °C for one month, most of the others stored in each condition for one, three and six months provided relatively weaker repellency (*χ*^2^ = 28.509, *df* = 9, *P* = 0.001) than the fresh sample. Furthermore, a lower repellency (*χ*^2^ = 14.624, *df* = 3, *P* = 0.002) was determined from AHE samples with longer storage time. It is plausible that extended storage times as well as fluctuating ambient temperature ranging from 21 to 35 °C, and high temperature of 45 °C, partially influenced either physical or biological stability of AHE materials. These findings corresponded to those of Turek & Stintzing [44], who suggested that monitoring volatile plant extracts and essential oil composition generally revealed forfeited stability from prolonged storage time as well as rises in temperature. These authors also reported that extrinsic parameters, particularly temperature, light and oxygen availability, affected stability of herbal products such as essential oils through oxidative and polymerization processes, with a loss of quality and pharmacological properties.

**Table 3:** Appearance and repellent activity (median complete protection time: Median CPT) of the fresh and stored samples of AHE against female *Ae. aegypti*

AHE samples (Temperature/Duration)		Appearance		Median CPT (Range, h)*
		Phase	Color	
Fresh sample		Liquid	Dark brown	6.50 (6.0–8.0) ab
Stored sample				
4 °C	1 month	Liquid	Dark brown	10.0 (8.0–11.0) c
	3 months	Liquid	Dark brown	7.25 (7.0–8.0) bd
	6 months	Liquid	Dark brown	6.0 (4.0–7.0) ade
Ambient temperature (21–35 °C)	1 month	Liquid	Dark brown	7.25 (6.5–8.5) acd
	3 months	Liquid	Dark brown	6.75 (4.0–7.0) ade
	6 months	Liquid	Very dark brown	4.5 (3.0–6.0) ef
45 °C	1 month	Liquid	Dark brown	5.75 (5.0–6.5) af
	3 months	Liquid	Very dark brown	4.5 (4.0–5.5) ef
	6 months	Liquid	Very dark brown	4.5 (4.0–5.0) ef

*Values followed by different letters in a column are significantly different (*P* < 0.05)

Surprisingly, AHE samples kept at 4 °C for one month afforded the median complete-protection time of 10.0 (8.0–11.0) h, which was extremely longer (*χ*^2^ = 12.722, *df* = 2, *P* = 0.002) than those of the fresh sample (6.50, 6.0–8.0 h). Although these outcomes cannot be explained herein, due to the absence of supportive experimental evidence, whether or not they resulted from low storage temperature is of interest. Turek & Stintzing [45] reported the strong stability of rosemary oil when kept at low temperatures, such as in the refrigerator, which could prevent oxidative reactions during three months of storage experiments. However, primary oxidation occurred in pine oil at only 5 °C, despite being promoted at 23 °C. Conversely, this reaction developed especially in lavender oil stored at 5 °C, when compared to that in room temperature, while it had almost degraded completely in both oils of pine and lavender at 38 °C [45]. Consequently, Turek & Stintzing [44] concluded that, based on their work and literature review, essential oils vary in susceptibility to autoxidation at different storage temperatures. They also suggested that analytical methods should be evaluated to assess both original and altered essential oil profiles with respect to their suitability for tracking chemical alterations. Analyzing chemical constituents in stored and fresh samples of AHE was, therefore, useful in manifesting not only bioactive substances responsible for repellency, but also chemical alterations affecting stability. This study was satisfied that all stored AHE samples achieved adequate protection times (4.5–10 h), which exceeded the minimum requirement (2 h) of the Food and Drug Administration (FDA) for sale in Thailand. Furthermore, AHE samples kept at ambient temperature for all durations serve as sufficient repellency (4.5–7.25 h) that is relatively close to that of the fresh sample. It is likely that AHE products can be kept in an ambient environment, which makes them convenient and practical in use and maintenance. However, the optimal storage conditions of this material are still low temperatures, such as those in a refrigerator. Further research directed at enhancing the stability of AHE is needed.

### Field repellent activity

In the preliminary field trials, a total of 1,339 adult female mosquitoes, comprising five genera, i.e. *Aedes*, *Anopheles*, *Armigeres*, *Culex* and *Mansonia*, were caught during the surveys. The most predominant mosquito genera were *Culex* and *Armigeres*, followed by *Aedes*, *Anopheles* and *Mansonia*, which totaled 727 (54.3 %), 580 (43.3 %), 24 (1.8 %), 4 (0.3 %) and 4 (0.3 %), respectively. Based on these findings, the large and mixed mosquito populations were considered sufficiently abundant for repellency evaluation. During the preliminary trials, sunset occurred at the testing site at around 19:30 h local time, and the mosquitoes gathered around 60 min before and after sunset, with the maximum mean collecting rate of 30.72 ± 13.2 (19:12–19:32 h). After that, the number of mosquitoes decreased gradually, but sufficient numbers were left for testing, with the minimum mean collecting rate of 19.94 ± 9.5 (21:02–21:22 h). Additionally, some mosquito species, particularly *Anopheles* and *Mansonia*, were collected mostly around 30 to 90 min after sunset. This information was then applied to fixing the optimal period for testing and collecting mosquitoes and the period between 18:00 and 21:30 h was deemed to provide the best chance of being bitten.

**Fig. 3:**
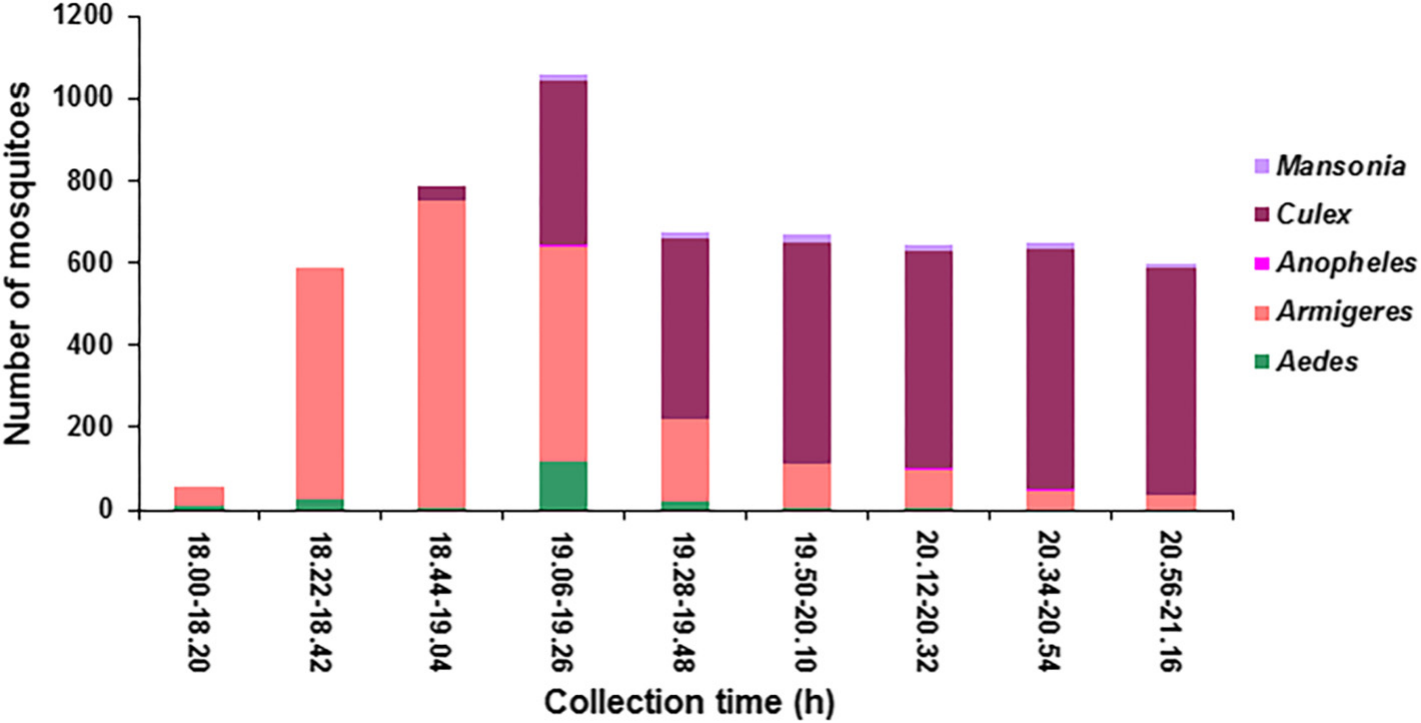
Distribution of mosquito species collected during the field repellent bioassays at Sunpesua subdistrict, Muang district, Chiang Mai Province, northern Thailand

The results of the field applications performed by human-baited techniques against the local mosquito populations are illustrated in Tables 4, 5 and Fig. 3. We found that varying species and numbers of mosquitoes were collected from the control volunteers only. Therefore, a highly significant difference (*χ*^2^ = 24.648, *df* = 2, *P* = 0.000) between the mean number of mosquitoes collected on the controls and testers treated with 25 % AHEv or 25 % DEETv was observed at every collecting site (CS); nine 20-min collections at each experiment (Table 4). From CS1 to CS4, the mean collecting rates of mosquitoes from the control volunteers increased dramatically (*χ*^2^ = 3.000, *df* = 3, *P* = 0.392). After that, the rates reduced moderately at CS5–CS9, but were still rather high. The maximum mean collecting rate was that of CS4, which was conducted between 19:06 and 19:26 h. These findings were consistent with guidance from the preliminary surveys, in that the mosquito collection period was suitable, due to the populous and mixed mosquitoes collected, which were abundant and available for calculating repellency.

**Table 4:** Number of mosquitoes and mosquito collecting rates (Mean ± standard error, SE) captured from human volunteers during field repellent bioassays at Sunpesua subdistrict, Muang District, Chiang Mai Province, northern Thailand

Collecting site (CS): Time	Treatment	No. of mosquitoes collected	Mosquito collecting rate (Mean ± SE)*
CS 1: 18:00–18:20 h	Control	56	2.0 ± 3.5 a
	25 % AHEv	0	0 b
	25 % DEETv	0	0 b
CS 2: 18:22–18:42 h	Control	588	21.0 ± 17.8 a
	25 % AHEv	0	0 b
	25 % DEETv	0	0 b
CS 3: 18:44–19:04 h	Control	787	28.1 ± 22.4 a
	25 % AHEv	0	0 b
	25 % DEETv	0	0 b
CS 4: 19:06–19:26 h	Control	1,058	37.8 ± 20.8 a
	25 % AHEv	0	0 b
	25 % DEETv	0	0 b
CS 5: 19:28–19:48 h	Control	672	24.0 ± 8.5 a
	25 % AHEv	0	0 b
	25 % DEETv	0	0 b
CS 6: 19:50–20:10 h	Control	667	23.8 ± 9.6 a
	25 % AHEv	0	0 b
	25 % DEETv	0	0 b
CS 7: 20:12–20:32 h	Control	641	22.9 ± 10.1 a
	25 % AHEv	0	0 b
	25 % DEETv	0	0 b
CS 8: 20:34–20:54 h	Control	650	23.2 ± 13.4 a
	25 % AHEv	0	0 b
	25 % DEETv	0	0 b
CS 9: 20:56–21:16 h	Control	599	21.4 ± 10.8 a
	25 % AHEv	0	0 b
	25 % DEETv	0	0 b
Total	Control	5,718	204.2 ± 73.2 a
	25 % AHEv	0	0 b
	25 % DEETv	0	0 b

*Mean in each collecting site followed by different letters is significantly different (*P* < 0.05)

**Table 5:** Results obtained from field repellent assessment of 25 % AHEv and 25 % DEETv, undertaken at Sunpesua subdistrict, Muang District, Chiang Mai Province, northern Thailand

Mosquito species	Control	25 % AHEv		25 % DEETv	
	No. of mosquitoes captured (%)	No. of mosquitoes captured (%)	Protection (%)	No. of mosquitoes captured (%)	Protection (%)
Genus *Aedes*					
*Ae. vexans*	123 (2.15)	0 (0)	100	0 (0)	100
*Ae. aegypti*	4 (0.07)	0 (0)	nd	0 (0)	nd
*Ae. albopictus*	46 (0.80)	0 (0)	100	0 (0)	100
*Ae. lineatopennis*	17 (0.30)	0 (0)	100	0 (0)	100
Genus *Anopheles*					
*An. barbirostris*	12 (0.21)	0 (0)	100	0 (0)	100
Genus *Armigeres*					
*Ar. subalbatus*	2,352 (41.13)	0 (0)	100	0 (0)	100
Genus *Culex*					
*C. gelidus*	38 (0.66)	0 (0)	100	0 (0)	100
*C. vishnui*	602 (10.53)	0 (0)	100	0 (0)	100
*C. quinquefasciatus*	2,371 (41.47)	0 (0)	100	0 (0)	100
*C. tritaeniorhynchus*	67 (1.17)	0 (0)	100	0 (0)	100
Genus *Mansonia*					
*M. indiana*	49 (0.86)	0 (0)	100	0 (0)	100
*M. uniformis*	13 (0.23)	0 (0)	100	0 (0)	100
*M. annulifera*	24 (0.42)	0 (0)	100	0 (0)	100
Total	5,718 (100)	0 (0)	100	0 (0)	100

*Abbreviation*: nd, not determined, as few specimens of this species were captured

Regarding the results demonstrated in Table 5, it appeared that 25 % AHEv afforded remarkable repellency (*χ*^2^ = 21.447, *df* = 1, *P* < 0.0001), which was comparable to that of 25 % DEETv. No mosquito bites were observed on the volunteers treated with 25 % AHEv or 25 % DEETv throughout the testing periods of the field study. Therefore, it should be concluded that 25 % AHEv and 25 % DEETv produced similarly strong repellency by minimizing bites with a 100 % protection against a wide range of field mosquito populations.

A total of 5,718 adult female mosquitoes belonging to five genera, i.e. *Aedes*, *Anopheles*, *Armigeres*, *Culex* and *Mansonia*, were collected during the field trials. Among 13 mosquito species collected, the most prominent were *Culex quinquefasciatus*, *Armigeres subalbatus* and *Culex vishnui*, which made up 41.47, 41.13 and 10.53 %, respectively. These results corresponded to those of preliminary surveys, which presented *Culex* and *Armigeres* as the principal mosquito genera collected. However, these findings did not coincide with those obtained from an earlier study conducted at the same place by Tuetun et al. [29], at that time the dominant mosquito species collected were *Ae. gardnerii* (35.1 %), *C. tritaeniorhynchus* (29.2 %), *C. vishnui* (19.4 %), *Ae. lineatopennis* (5.1 %), *Ar. subalbatus* (3.8 %) and *M. uniformis* (2.3 %). The difference in mosquito populations collected almost a decade apart is likely a major consequence of environmental changes due to the extension of cities and towns. Noticeable changes in the site and surrounding areas of this study are a reduction of rice fields and gardens, with increasing sources of polluted water. It is generally known that *C. quinquefasciatus* and *Ar. subalbatus* breed profusely in sewage or polluted water deposits [46, 47], whereas *Ae. gardnerii* is found usually in tree holes, log holes, bamboo stumps and bamboo cups and *C. tritaeniorhynchus* is commonly seen in irrigated rice fields and ditches, as they prefer cleaner water [48, 49]. It becomes evident from the outcome of this study that *Ae. gardnerii* and *C. tritaeniorhynchus* populations have been replaced by *C. quinquefasciatus* and *Ar. subalbatus* in this location.

In this study, the maximum mean collecting rates of the dominant mosquito species, *Culex* and *Armigeres*, were observed in different periods (Fig. 3). *Ar. subalbatus* gathered in the evening before sunset with an activity peak at between 18:44 and 19:04 h before decreasing continually after sunset. By contrast, fewer *Culex* species were seen before sunset, but increased consecutively after it, with a biting peak between 20:34 and 20:54 h. A varied pattern in biting behavior of these mosquitoes was observed each day of the field collections. This possibly related to differences in feeding or biting behavior of each mosquito species. *Armigeres subalbatus* is a vicious crepuscular biter that frequently feeds at dusk and dawn, whereas *Culex* spp. respond negatively to light intensity and become active after sunset, when they mostly feed at night [49, 50]. These mosquitoes are associated closely with human habitations because of their anthropophilic and breeding areas [46, 51]. Despite there being no evidence of *Ar. subalbatus* transmitting pathogens to humans in Thailand, it has been shown as an efficient vector of the dog heartworm, *Dirofilaria immitis* [52]. *Culex quinquefasciatus* is presently regarded as an important vector of filariasis and Japanese encephalitis in the tropical and subtropical regions [53–55]. However, the risk from mosquito-borne diseases has not been reported in the area of the present study. One reason for this is probably that almost all inhabitants lived in houses with protection from insect bites such as screened doors and windows. In general, the feeding behavior of mosquitoes is a significant factor that determines whether they are important as nuisance insects or vectors of diseases, which governs the selection of control methods [49]. Species that prefer to feed on animals are inefficient at transmitting diseases from human to human. Those that feed in the early evening may be more difficult to avoid than those that feed at night. However, information on epidemiology and disease-vector relationships in the locality of this study is not available and warrants more extensive research such as surveys for larval habitats, mosquito collections and evaluation of local mosquito populations for the presence of pathogenic infections.

The complete protection of 25 % AHEv against the most abundant mosquitoes, *C. quinquefasciatus*, *C. vishnui* and *Ar. subalbatus* as well as other mosquito species, such as *Ae. vexans*, *Ae. albopictus*, *Ae. lineatopennis*, *An. barbirostris*, *C. gelidus*, *C. tritaeniorhynchus*, *M. indiana*, *M. uniformis* and *M. annulifera*, was considered to have significantly promising potential. However, the number of remaining *Ae. aegypti* (4; 0.07 %) collected was too small to allow a valid estimate of the protective level against this species. The low number of *Ae. aegypti* collected was possibly because either the testing period (between 18:00 and 21:30 h) was not concurrent with its prime biting time or the study site was not a suitable location for finding this mosquito species. Although 25 % AHEv presumably protects against *Ae. aegypti*, as proven in the laboratory, the insufficient number collected in this field experiment could not confirm repellency against the natural population. As *Ae. aegypti* is the most important vector of dengue fever in urban areas of Thailand [56, 57], further field studies should survey the optimal location and time period for repellent testing against its natural populations. Field studies using local mosquito populations not only provide and confirm an accurate potential of repellent against known mosquito pests and disease vectors, but also the results obtained are important when recommending repellent use to the public [5, 58].

No local skin reactions such as rash, swelling, irritation, hot sensation, or other allergic responses were observed in the subject volunteers during both laboratory and field study periods. The dermal toxicity of AHE has not been evaluated previously in either humans or animals. However, *A. sinensis* has been reported as herbal medicine formulated clinically to treat various forms of skin trauma and wounds [59, 60]. Evaluation on the pharmacological effects revealed that the ethanolic extracts of this plant contributed in the process of wound healing by effectively promoting skin fibroblast proliferation with low levels of cytotoxicity even at high concentrations [60]. Furthermore, this herbal extract was proven to have the therapeutic property on atopic dermatitis by inhibition of allergic and inflammatory mediators [61]. These findings supported the relatively safe application of this plant product on skin. Based on the experimental results obtained in this study, it has been proven that *A. sinensis* offers not only impressive repellency against laboratory *Ae. aegypti* and a wide range of natural mosquito populations, but also relative stability in physical and biological performance. Additional research with advanced formulation techniques and toxicity testing is needed to transform AHE-based repellents for exploitable commercial production.

## Conclusions

The remarkable repellency of AHE is comparable to DEET in both laboratory assessments with *Ae. aegypti* and field applications against a wide range of natural mosquito populations, and has proven potential for being transformed for exploitable commercial production. AHE-based repellents with profound efficacy and pronounced stability produced by advanced formulation techniques, particularly nanotechnology, are the principle of further studies. Additionally, in order to reach manufacturer and customer acceptability, parameters relevant to health, cosmetics, and economical aspects must also be considered.

## Abbreviations

AHE, *Angelica sinensis* hexane extract; AHEv, *Angelica sinensis* hexane extract +5 % vanillin; ANOVA, analysis of variance; CMU, Chiang Mai University; CS, collecting site; DEETv, DEET +5 % vanillin; EI, electron ionization; GC-MS, gas chromatography-mass spectrometry; Median CPT, median complete protection time; RT, retention time; WHO, World Health Organization
